# African swine fever vaccines: a promising work still in progress

**DOI:** 10.1186/s40813-020-00154-2

**Published:** 2020-07-02

**Authors:** Laia Bosch-Camós, Elisabeth López, Fernando Rodriguez

**Affiliations:** IRTA, Centre de Recerca en Sanitat Animal (CReSA, IRTA-UAB), Campus de la Universitat Autònoma de Barcelona, 08193 Bellaterra, Spain

**Keywords:** African swine fever (ASF), African swine fever virus (ASFV), Live attenuated virus (LAV), Subunit vaccines, Antibodies, Cytotoxic CD8^+^ T-cells (CTLs), Protection, Biosafety

## Abstract

**Abstract:**

African swine fever (ASF), a disease of obligatory declaration to the World Organization for Animal Health (OIE), has contributed to poverty and underdevelopment of affected areas. The presence of ASF has been historically neglected in Africa, contributing to its uncontrolled expansion and favouring its spread to continental Europe on at least three occasions, the last one in 2007 through the Republic of Georgia. Since then, African swine fever virus (ASFV) has spread to neighbouring countries, reaching the European Union in 2014, China in the summer of 2018 and spreading through Southeast Asia becoming a global problem. Lack of available vaccines against ASF makes its control even more difficult, representing today the number one threat for the swine industry worldwide and negatively affecting the global commerce equilibrium.

**Main body:**

In this review, we intend to put in perspective the reality of ASF vaccination today, taking into account that investment into ASF vaccine development has been traditionally unattractive, overall since ASF-free areas with large swine industries applied a non-vaccination policy for diseases listed by the OIE. The dramatic situation suffered in Asia and the increasing threat that ASF represents for wealthy countries with large swine industries, has dramatically changed the perspective that both private and public bodies have about ASF vaccinology, although this is controversial. The feasibility of modifying the ASFV genome has led to safe and efficacious experimental recombinant live attenuated viruses (LAVs). The main challenge today will be confirming the safety and efficacy of these technologies in the field, accelerating transfer to the industry for official registration and commercialization. The complexity of ASFV, together with the lack of knowledge about the mechanisms involved in protection and the specific antigens involved in it, requires further investment in research and development. Although far from the efficacy achieved by LAVs, subunit vaccines are the optimal choice for the future. If the world can wait for them or not is a contentious issue.

**Conclusion:**

Despite their inherent disadvantages, LAVs will be the first technology to reach the market, while subunit vaccines will need much further research to become a successful commercial reality.

## Background

African swine fever (ASF) is a swine hemorrhagic viral disease that is of mandatory declaration to the World Organization for Animal Health (OIE). The disease is caused by African swine fever virus (ASFV), a large nucleocytoplasmic virus that contains a double-stranded 170–190 kb DNA genome, encoding 151–167 open reading frames (ORFs) [1]. The ASFV virion is a complex multi-enveloped and multi icosahedral particle containing at least 68 different structural viral polypeptides and 21 cellular proteins and possess a much more complex structure than previously believed [1–3]. ASFV replicates predominantly in mononuclear-phagocytic cells (monocytes and macrophages), which play a front-line role in activating and orchestrating the innate and adaptive host responses. During infection, ASFV express multiple proteins that subverts several host pathways in order to ensure efficient virus replication in vivo [4]. ASF was described for the first time in Kenya in 1921 as a lethal hemorrhagic disease affecting the first European imported domestic pigs and being maintained asymptomatically in a sylvatic cycle between ticks of the genus *Ornithodoros* and warthogs (*Phacochoerus* spp.), acting as long-term carriers and reservoirs of ASFV [5]. The complex live cycle of ASFV in this region explains the actual presence of more than 24 different ASFV genotypes, classification based on the sequence of B646L, encoding p72, the major capsid protein [6]. Outside Africa, only genotypes I and II have been found and both domestic pigs and wild boars (both being *Sus scrofa*), are equally susceptible to the disease [7]. When introduced into disease-free regions or domestic pig populations, the disease predominantly shows acute forms with high mortality rates up to 100%. After several years of ASFV presence in endemic areas, mortality rates decline due to virus adaptation to the hosts and infected individuals show subacute forms of the disease or even no clinical signs, complicating even more its detection and eradication, as described in the Iberian Peninsula [8]. ASFV can spread into neighboring areas carried by free-ranging infected wild animals but it can also emerge into new distant areas by the illegal transport of infected pigs, the use of contaminated pork or pork products for feeding pigs, or even fomites such as equipment (boots, trucks, etc.) not properly disinfected. Once established, the virus disseminates via oral-nasal routes by direct contact between infected and non-infected individuals or with contaminated products [9]. Unfortunately, ASFV is one of the most resistant viruses to chemical and physical inactivation, therefore complicating the tasks of disinfection. Transcontinental spread of ASFV from Africa first occurred in two consecutive waves: 1957 and 1960, when genotype I viruses emerged in Spain and Portugal, where it became endemic and spreading to other European countries, South America and the Caribbean. The eradication campaign from the European Continent lasted more than 30 years and provoked enormous economic losses in affected countries [8]. The complex ASFV epidemiological situation above described in Africa, together with the absolute lack of intervention from the rest of the world, facilitated the exportation of the virus again to Europe, catching us totally off guard and unprepared. Thus, barely 10 years after declaring the continental European Union (EU) free of ASF, a second transcontinental outbreak occurred in 2007, when genotype II ASFV from Eastern Africa was first detected in Georgia [10]. From the Caucasus, and with the involvement of wild boars in the spread, ASFV broke first into Eastern Europe and Baltic Countries, reaching the first EU countries in 2014. In August–September 2018, ASFV was declared in China, the country that raises half of the world’s pig population and a year and a half later the situation can be described as a global epidemic threating global swine production [11].

### The need of a vaccine for ASFV

Lack of vaccines against ASF leaves the control of the disease to an efficient and rapid diagnosis and the culling of infected and exposed animals (www.oie.int). This strategy has been demonstrated as inefficient in countries with limited resources, incapable of implementing fair policies of compensation and ASF control.

Lack of efficient treatments or vaccines against ASF complicate ASF control even more. Development of vaccines against ASF has been historically unattractive for the industry. On one hand, ASF remained endemic almost exclusively in Africa, and on the other hand, ASF-free countries with developed swine industries apply a non-vaccination policy for diseases of obligatory declaration to the OIE (www.oie.int). Therefore, there is still a lot of work to perform to provide safe and efficient vaccines and to guarantee their optimal application in the field [12]. However, the entrance of ASFV in China has totally changed the perception of ASF vaccinology [13, 14]. Limited resources together with the complexity of the virus have contributed to explaining the gaps existing today regarding ASFV infection and immunity. So far, the precise nature of the host protective responses has not been fully determined and protective antigens remain to be identified, hampering the rational design of vaccines. Despite these limitations, the few laboratories devoted to work on ASF vaccinology, have contributed to several seminal findings in the field [15]. Thus, we can confirm today: first, that vaccines against ASF are not a utopia; second, that LAVs can be a short-medium term reality to fight the disease, overall in endemic regions or regions in danger to become endemic; and third, that subunit vaccines can be a long-term vaccine choice.

Collaborative efforts between academia and industry should allow the improvement of safety and DIVA properties (capable to Differentiate Infected from Vaccinated Animals) of the experimental vaccine prototypes below described. This should be the beginning of a new era for ASF vaccinology where continuous collaborative research will be needed to obtain the safest and most efficient vaccine in the future. Controlling the virus in Asia and Europe is mandatory for the swine industry, but controlling ASFV in Africa should be mandatory for humanity. Even if we do not appeal to humanitarian reasons, we should turn to common sense, since reducing the epidemiological pressure of ASFV in Africa should diminish the risk of exportation of new ASFV strains in the future. Collaboration between researchers in the field, private companies, governmental bodies and international organisms such as the OIE and the Food and Agriculture Organization of the United Nations (FAO) is necessary if we are willing to succeed in controlling ASF, today the number one global threat for animal health.

#### Classical vaccines: understanding the mechanisms involved in protection

Classical vaccines based on inactivated ASFV, independently of the inactivation method and the adjuvant used, have proven ineffective [16, 17]. Despite inactivated vaccines being very efficient at inducing antibodies, on occasions capable of blocking the virus in fluids, they are not very efficient at inducing specific cytotoxic CD8^+^ T-cells (CTLs) [18], crucial for elimination of virus-infected cells. Incorporation of new adjuvant formulations and novel and more innocuous inactivation procedures, might contribute to designing efficient inactivated vaccines in the future [1–3].

LAVs, whether naturally attenuated or adapted to tissue culture, have been used as experimental models to understand the mechanisms involved in homologous protection against ASF.

The presence of ASFV-specific CTLs was initially demonstrated using PBMCs from animals surviving the infection with attenuated viruses [19–21]. More recently, the CTL activity has been associated to both CD8^+^ and double positive CD4^+^/CD8^+^ T-lymphocytes, the latter thought to include memory/effector T cells [22]. The definitive evidence identifying ASFV-specific T-cells as crucial for ASFV protection came after the demonstration that in vivo depletion of CD8^+^ cells abrogated protective immunity to ASFV [23]. Today we know that two distinct phenotypes of porcine CTLs are capable to lyse ASFV-infected cells: a conventional CD8 single positive, and a CD4^+^CD8αβ^+^ phenotype, both being perforin positive and showing swine leukocyte antigen I (SLA I)-restricted cytotoxicity. Increasing levels of circulating conventional CD8^+^ T-lymphocytes have been correlated with the onset of ASF clinical signs, while CD4/CD8αβ double positive CTLs were significantly increased in protected pigs [24].

Similarly, passive administration of antibodies or colostrum from ASF convalescent pigs, conferred partial protection against experimental ASFV lethal challenge [25, 26], therefore demonstrating their protective potential. Nonetheless, 25 years later, the exact mechanisms by which this partial protection was afforded are not yet fully understood. While some authors correlated the protection afforded with the presence of antibody-dependent cell-mediated cytotoxicity [26], others associated it with the presence of neutralizing antibodies [27], an issue still controversial today [28]. A third complementary mechanism of antibody-mediated protection has been postulated [29] based on studies using sera from pigs surviving the infection with several hemadsorbing ASFV strains. In these studies, a direct correlation was observed between the presence of hemagglutination inhibitory antibodies, the ability of these antibodies to in vitro inhibit ASFV infection and the capability of these animals to survive the experimental challenge with the homologous virulent virus [30].

In our vision, both an adequate innate immune response [31] together with adaptive responses, including antibodies and CD8^+^ T cells, will be essential to induce sterilizing immunity against ASFV.

Despite their enormous efficacy, the use of ASF LAVs as vaccines in the field is still controversial [12], mainly due to biosafety concerns related to their inherent infectious nature. Natural attenuation of ASFV in endemic areas has complicated its eradication, mainly due to the difficult identification of chronically infected pigs that might act as continuous reservoirs [8]. ASFV chronicity paralleled with a total change of clinical findings [32]. The concomitant presence of virus and antibodies correlated with the presence of lesions compatible with immune complex deposition, including necrotic foci in joint swelling [33–35]. Antibody exacerbation phenomenon has been also observed after immunization with inactivated virus and even after experimental immunization with subunit vaccines [17, 36–38]. The fine balance existing between the protective and detrimental role of ASFV-specific antibody response needs further investigation, both from the basic and applied point of view. Understanding the intrinsic mechanisms explaining the protection and the exacerbation caused by ASFV-specific antibodies is crucial for future vaccine designs. Albeit not frequently reported, natural attenuation of ASFV has been also reported more recently in the Caucasian region, mostly associated to wild boars [39, 40]. Experimental infection with some of these new isolates ratified their attenuation in domestic pigs [40] and in wild boars [41]. Together with the polemic use of these ASFV strains as vaccines or as vaccine templates in the future [12, 41], their presence in nature open new concerns about the eradication measures today in place, based on detecting ASFV nucleic acid. The presence of attenuated ASFV isolates in the field deserves deeper studies and if confirmed, evaluating the additional implementation of antibody detection methods as performed during the eradication campaigns in Spain [42]. China, where the virus has infected more pigs in only 1 year that in its entire history, might parallel the fastest evolution ever recorded for ASFV, therefore increasing the risk for the region to become endemic. What in the short term might look like a successful exit from the current living drama could bring long-term consequences for global commerce.

#### Recombinant LAVs: coming closer to the market

Today the possibility of deleting specific genes using recombination technologies [43] or by genome editing using the CRISPR-Cas9 system [44], opens the possibility of obtaining safer LAVs. The main LAVs tested in vivo are summarized In Table 1*,* especially remarking those with potential to protect against the virus currently circulating in Europe and Asia. Genome manipulation has provided crucial information about essential and non-essential ASFV genes involved in replication, viral morphogenesis and of course, in virus virulence [4, 65].

**Table 1:** Recombinant LAVs tested in vivo, specially indicating those providing protection again the Genotype II virus currently circulating in Asia and Europe (bolded data)

Deleted gene(s)	Protein(s)	Parental ASFV		Recombinant LAVs		Target cells	Reference
		Strain	Virulence	Virulence	Protection		
**EP402R**	**CD2v**	BA71V	Non-pathogenic	Non-pathogenic	None	Vero	[45]
		Malawi Lil-20/1	Virulent	Virulent	None	Macrophages	[46]
		Georgia2010	Virulent	Virulent	None	Macrophages	[47]
		**BA71**	**Virulent**	**Attenuated**	**BA71, E75, Georgia2007/1**	**COS-1 & Macrophages**	[48]
DP71L	NL	Malawi Lil-20/1	Vrulent	Virulent	Malawi	Macrophages	[49, 50]
		E70	Virulent	Attenuated	E70	Macrophages	[50, 51]
**B119L**	**9GL**	Pretoriuskop/96/4	Virulent	Attenuated	Pretoriuskop/96/4	Macrophages	[52]
		Malawi Lil-20/1	Virulent	Attenuated	Malawi	Macrophages	[53]
		**Georgia2010**	**Virulent**	**Attenuated**	**Georgia2010**	**Macrophages**	[54]
DP96R	UK	E70	Virulent	Attenuated	E70	Macrophages	[55]
DP148R		Benin	Virulent	Attenuated	Benin	Macrophages	[56]
**MGF360 & MGF505**		Benin	Virulent	Attenuated	Benin	Macrophages	[57]
		**Georgia2010**	**Virulent**	**Attenuated**	**Georgia2010**	**Macrophages**	[58]
**B119L & DP96R**	**9GL & UK**	**Georgia2010**	**Virulent**	**Attenuated**	**Georgia2010**	**Macrophages**	[59]
MGF360 & MGF505 & B119L		Georgia2010	Virulent	Highly attenuated	None	Macrophages	[60]
B119L & DP96R & DP71L	9GL & UK & NL	Georgia2010	Virulent	Highly attenuated	None	Macrophages	[61]
DP71L & DP96R	NL & UK	OUR T88/3	Attenuated	Highly attenuated	None	Macrophages	[62]
A238L A224L EP153R	NFAT regulator Apoptosis inhibitor MHC-I antigen presenting modulator	NH/P68	Attenuated	Highly attenuated	L60	COS-7 & Macrophages	[63]
**MGF360 & MGF505 & EP402R**	**MGF505-1R, MGF505-2R, MGF505-3R, MGF360-12 L, MGF360-13 L, MGF360-14 L & CD2v**	**China HLJ/18**	**Virulent**	**Attenuated**	**China HLJ/18**	**Macrophages**	[64]

As a good example for the purpose of this review, depletion of EP402R gene from the non-pathogenic BA71V isolate, allowed in vitro identification of CD2v (EP402R product), as the ASFV hemagglutinin [45]. Unfortunately, the non-infectious nature of BA71V made the in vivo characterization of BA71V∆CD2, or any other recombinant virus made on this background impossible [66]. The first deletion mutant tested in vivo was ∆8-DR, a recombinant precisely lacking CD2v, made on the virulent Malawi Lil-20/1 ASFV strain [46]. Despite its incapability to bind red blood cells, ∆8-DR was still lethal in vivo*,* showing a delay/reduction in viremia kinetics but causing clinical signs and mortality similar to those caused by the parental virus. From here, few genes have been identified as implicated in the virulence of different ASFV virulent strains: DP71L (NL), B119L (9GL), DP96R (UK), DP148R and multigene families 360 and 505 (MGF360 & MGF505) [49–53, 55–57]. Some of these genes have been also eliminated from Georgia2010, the Genotype II virus currently circulating in Europe and Asia; deletions that have caused attenuation and different degrees of homologous protection in vivo [54, 58]. Interestingly, the concomitant elimination of the 9GL and UK from the Georgia2010 virus resulted in more effective vaccine prototypes than those lacking individual ORFs [59]. Similarly, recent results published in Science China Life Science, demonstrate the safety and the efficacy of a new recombinant LAV obtained from deleting the already described MGF360/530 and CD2v virulence factors [64]. It is of crucial relevance for beginners in the field to be aware that concomitant elimination of ASFV virulence factors does not always result in better attenuation, on occasions yielding very weak viruses incapable to grow in vivo*,* or at least incapable of inducing protective responses. This is the case for example of a Georgia2010 triple mutant lacking 9GL, UK and NL [61] or a Georgia2010 double mutant lacking 9GL and MGF360/MGF505 [60]. Likewise, depletion of specific virulence factors from natural LAVs, have decreased their ability to protect against challenge with the parental virulent virus [62, 63]. And finally, depletion of a single gene can result in different phenotypes depending on the virus strain used, as has been demonstrated for NL [51], and more recently for CD2v [47, 48]. These observations are of extreme relevance when rationally designing LAVs against ASFV, since on most occasions they limit their protection against the parental virulent strain (homologous virus) but not against heterologous strains. The definition of homologous and heterologous for ASF has not been well defined, observing a lack of cross-protection on occasions between viruses isolated in close endemic regions and in the same period [30]. Although useful to understand ASFV evolution, genotyping [6] does not correlate with protection. On the other hand, serotyping based on hemadsorbing inhibition assays, seems to partially explain cross-protection [67], but there is still much to learn, since protection can be afforded in the absence of CD2v [48, 62, 63]. Furthermore, viruses with identical CD2 and lectin behave as heterologous in vivo [30]. Lack of in vitro correlates for protection leaves experimental in vivo challenging as the only way to demonstrate the cross-protective nature of a novel vaccine.

Twenty-five years after its deletion from the non-pathogenic BA71V [45], the EP402R gene (CD2v) was deleted from the parental BA71 virulent virus to obtain BA71∆CD2 for the completion of in vivo studies [48]. BA71∆CD2 induced strong humoral and cellular responses and conferred solid protection against the homologous virus (BA71) in a dose-dependent manner. For the first time solid protection was conferred not only against homologous virus but also against heterologous viruses such as E75 (Genotype I) or the phylogenetically more distant Georgia2007/1 strain (Genotype II) currently circulating in Continental Europe and Asia. Finally yet importantly, BA71∆CD2 presents the additional advantage of being capable of stably growing in the commercial Cos-1 cell line without needing previous adaptation. Despite the impressive protective capacity achieved with this experimental vaccine, BA71∆CD2 needs further improvement, mainly from the biosafety point of view. A small proportion of vaccinated pigs show short periods of low, albeit detectable BA71∆CD2 in both sera and nasal swabs coinciding with mild fever spikes, but no other ASF-compatible clinical signs. All animals end up eliminating the virus in the first 4 weeks after vaccination.

This chapter deserves a closing with unexpected good news. The specific deletion of the previously uncharacterized I177L gene provokes the dramatic attenuation of the virus and most importantly, confers sterilizing protection against the virus currently threating our pig industry [68]. This result clearly demonstrates that the systematic deletion of each one of the ASFV genes, independent of in silico predictions could bring satisfactory surprises. This is not a question of luck but conversely, rewards one of the few groups consistently working on ASFV vaccine discovery, a field with plenty of frustrations that also bring some satisfactions.

Despite their imperfections, LAVs available today confer a level of protection against experimental ASF infection far better than any other vaccine strategy so far tested (see the following chapter). Ideal LAVs should however accomplish the following requisites before becoming a reality in the field:

1. **Safety**. Experimental LAVs should demonstrate in the field not only to be efficacious, but also safe for the vaccinated pigs, for the in-contact animals and for the environment.
2. **DIVA.** Vaccinated pigs should ideally induce an immune response distinguishable from naturally infected pigs.
3. **Biofactory.** Vaccines should be produced under high quality standards and following the policy of the corresponding regulatory agencies, thus requiring stable cell lines for producing the LAV at a large scale.
4. **Cross-protection.** Cross-protective vaccines could be useful not only to protect a specific region against a single virus, but against many different viruses from the same genotype or from different genotypes (ideally all), thus covering endemic regions of Africa where up to 24 genotypes have been described.
5. **Wildlife**: LAVs formulations compatible with wild-life immunization campaigns will also be demanded if willing to control and eradicate ASF. Bait-vaccine formulations should also demonstrate their safety and efficacy in field studies. 

Vaccines are one of the best human inventions, but this truth circumscribes exclusively the “good ones”: those proven efficient, safe and correctly administered in the field. Balancing the risks and the benefits of implementing each available vaccine, applying scientific criteria and avoiding pre-established interests and prejudices, should be mandatory in the future.

### Antigen discovery and development of subunit vaccines

In order to avoid, or at least diminish, the unwanted side effects of traditional inactivated and attenuated vaccines, subunit vaccines arise as an ideal option for the future development of ASF vaccines [15]. As mentioned above, little is known about the mechanisms involved in protection and even less about the antigens responsible of such protection. To fill these gaps, research from both public administrations and the private sector should be encouraged: from antigen discovery to adjuvant and expression vector, more research investment is needed to design the ideal vaccines of the future. Screening of potential antigenic determinants within the ASFV is a challenging task due to the complex nature of this virus, encoding more than 150 ORFs and from which many of them there is neither expression nor functional data available. In this section, we collect relevant studies regarding ASFV antigen discovery as well as significant advances in the development of subunit vaccines against ASF.

#### The Yin and the Yang of ASFV-specific antibodies

The ASFV serological determinants determined so far are listed in Table 2, specifically indicating those capable of inducing antibody responses after immunization in vivo. As described above, the presence of neutralizing antibodies and their potential role in protection is still today polemic [28]. On the one hand, neutralizing monoclonal antibodies recognizing the major capsid protein p72 were described decades ago [76], but no further development has been recorded. Similarly, specific polyclonal antibodies against p54 (encoded by the E183L ORF) and p72 (B646L) were capable of blocking the ASFV-attachment to susceptible cells, while antibodies against p32 (CP204L) inhibited virus internalization, both mechanisms essential to protect pigs against E75 lethal challenge [27]. Unfortunately, the protection afforded by p72, p54 and p32 was not reproducible using other infectious model systems [77], opening new concerns about their relevance in protection and contributing to feed the controversy still in force, about the protective role of antibody-mediated neutralization. Due to the fact that p72, p32 and p54 are among the most immunogenic structural proteins, they have been included in diverse experimental vaccine formulations aiming to confer protection against several virulent ASFV isolates, with little if any significant effect [37, 38, 72, 73, 77].

**Table 2:** ASFV serological immunodeterminants, indicating those described to be recognized by sera from pigs recovered from ASFV infection (antigenic) or to induce immune responses after in vivo immunization (immunogenic)

Gene name	Protein	Antigenic	Reference	Immunogenic	Reference
**Structural**					
A104R	Histone-like DNA-binding protein	Yes	[69, 70]	Yes	[71]
A137R	P11.5	–	–	Yes	[72]
B438L	p49	–	–	Yes/Low	[73, 74]
B602L	p72 chaperone	Yes	[69, 70]	Yes	[74, 75]
B646L	p72, major capsid protein	Yes	[27, 69, 76]	Yes	[71, 72, 77, 78]
CP204L	p30	Yes	[27, 69]	Yes	[38, 71–73, 77–79]
CP2475L	pp220	–	–	Yes	[72]
CP2475L/partial	p37	–	–	Yes	[71]
CP530R	pp62	Yes	[80]	Yes	[38, 81]
CP530R/partial	p35	–	–	Yes	[37, 78]
CP530R/partial	p15	–	–	Yes	[37, 78]
D117L	Major transmembrane protein p17	–	–	Yes	[73]
E183L	p54	Yes	[27, 69, 70]	Yes/Low	[37, 71, 73, 77–79]
EP153R	C-type lectin	–	–	Yes	[73]
EP402R	HA/CD2v	Yes	[82]	Yes	[71, 73, 78]
K78R	p10, DNA-binding	Yes	[69]	–	–
KP177R	p22	–	–	Yes/Low	[73, 77]
E120R	p14.5	–	–	Low	[73]
H108R	Inner envelope	–	–	Low	[73]
O61R	p12	–	–	Yes	[73]
**Non-structural**					
B119L	9GL, virus assembly	–	–	Yes	[74]
F334L	RNA reductase	Yes	[69]	–	–
I215L	Ubiquitin conjugating enzyme	–	–	Yes	[38]
K196R	Thymidine kinase	Yes	[69]	–	–
L10L	KP177R-related	–	–	Yes	[73]
NP419L	DNA ligase	Yes	[69]	–	–
**Uncharacterized**					
A151R	–	–	–	Yes	[74]
C129R	–	–	–	Yes	[38]
C44L	–	Yes	[69]	–	–
CP312R	–	Yes	[69]	Yes	[78]
E184L	–	Yes	[69]	–	–
K145R	–	Yes	[69]	–	–
K205R	–	Yes	[69, 70, 75]	Yes	[74]
M448R	–	–	–	Yes	[38]
**Multigene families**					
MGF110-4 L	XP124L	–	–	Yes	[38]
MGF110-5 L	V82L	–	–	Yes	[38]

Parallel studies performed aiming to identify ASFV proteins responsible of inducing hemagglutination inhibitory antibodies [30], provided the identification of CD2v as the ASFV hemagglutinin. Thus, immunization of pigs with Sf9 insect cells infected with a recombinant baculovirus encoding the ASFV protein CD2v in the presence of Freund’s adjuvant, conferred protection against E75 ASFV challenge. The protection afforded was dose-dependent and correlated with the presence of antibodies capable of inhibiting hemagglutination and the homologous ASFV infection in vitro [29]. This work has been more recently confirmed by identifying CD2v and the ASFV lectin as key protective antigens [83], which could be useful to serotype ASFV [67]. As described for p32, p54 and p72, some experimental vaccine formulations containing CD2v have failed at inducing solid protection, perhaps reflecting antigenic and pathogenic differences between the ASFV strains used or in the vaccine formulations per se [37, 38, 78, 81, 84].

Together with p54, p32 and p72, many other antigens have been identified by sera from recovered pigs as immunodominant antigens, including the structural polyprotein pp62 [80]. Sera from convalescent pigs also recognized some other structural proteins (A104R, C-type lectin pEP153R, chaperone pB602L and K78R), non-structural proteins (RNA reductase, DNA ligase and thymidine kinase) as well as proteins of unknown function and location (C44L, CP312R, E184L, K145R and K205R) [69, 70, 75, 85]. Unfortunately, the protective efficacy of most of these antigens has not been confirmed.

More recently, immunization of pigs with complex ASFV-antigen formulations induced specific antibody responses that allowed the identification of additional antigenic determinants. Thus, Babraham syngeneic pigs immunized with a cocktail of 44 antigens in a DNA-prime recombinant vaccinia-boosting regime, recognized D117L as a novel ASFV immunogenic protein [73]. After challenge with Georgia2007/1, all pigs developed acute ASF, albeit viral genome levels were significantly reduced in immunized pigs in blood and lymphoid tissues. More recently, adenovirus-priming and vaccinia Ankara (MVA)-boosting regimes using diverse antigen combinations, allowed the definition of five additional ASFV antigens as good antibody inducers: MGF110-4 L, MGF110-5 L, M448R, C129R, I215L [38]. As before, immunization did not protect pigs from acute ASF (OUR T88/1 challenge) but reduced the viremia in a proportion of outbred pigs.

Immunization with different cocktails of recombinant adenoviruses allowed the confirmation of the immunogenicity of B602L, K205R-A104R, CP530R, CP2475L and the extracellular domain of EP402R, identifying for the first time A151R and B119L as good antibody inducers, albeit none of the cocktails induced significant protection against Georgia2007/1 [71, 74, 81]. Immunization with recombinant adenoviruses encoding: p32, p54, pp62, p72, and p37 and p150 (mature proteins processed from the pp220 polyprotein) seemed to afford some protection depending on the adjuvant used, while other vaccine formulations exacerbated the disease [81].

Despite the efforts made, none of these antigen-specific antibodies have been confirmed to be protective in an unarguable manner and we are still far from identifying ASFV antigens capable to confer solid protection against Georgia2007/1 challenge and even further from the optimal formulations. In fact, antigenicity and protection do not concur and, on occasions, and an inverse correlation has even been demonstrated. This effect has been described upon natural and experimental ASFV infection with LAVs and also after immunization with inactivated and subunit vaccine formulations [17, 36–38, 81].

The fine balance existing between the protective and detrimental role of ASFV-specific antibody response needs further investigation, both from the basic and applied point of view. Antibodies play a role in protective immunity against ASFV, although the specific antigens and/or the way to be targeted are still unknown.

#### The unknown: ASFV antigens inducing T-cell responses

ASFV vaccinology, as well as traditional veterinary vaccinology in general, has focused on triggering antibody responses, while little attention has been paid to the role that cellular responses play in protection. This reality has dramatically changed in recent years, and future vaccinologists would have to focus their attention also on this crucial arm of the immune response. However, few in vitro studies have centered their attention on identification of ASFV proteins inducing CTLs. The antigens so far recognized by ASFV-specific T-cells, as well as those described as immunogenic in vivo (capable to induce specific T-cell responses upon immunization), are listed in Table 3.

**Table 3:** ASFV-specific T-cell determinants, indicating those described to be recognized by PBMCs from pigs recovered from ASFV infection (antigenic) and those inducing T-cell responses after in vivo immunization (immunogenic)

Gene name	Protein	Antigenic	Reference	Immunogenic	Reference
**Structural**					
B602L	p72 chaperone	–	–	Yes	[73]
B646L	p72	Yes	[38, 86]	Yes	[38, 71, 73]
CP204L	p30	Yes	[38, 87]	Yes	[38, 71, 73]
CP2475L	pp220	–	–	Yes	[73]
CP530R	pp62	Yes	[38]	Yes	[38, 71, 73]
CP530R/partial	p15	–	–	Yes	[37]
D117L	–	–	–	Low	[73]
E183L	p54	–	–	Yes	[71, 73]
E248R	Transmembrane myristoylated protein	Yes	[38]	–	–
EP153R	C-type lectin	Yes	[88]	Yes	[73]
EP402R	HA/CD2v	Yes	[88]	Yes	[73, 89]
H108R	Inner envelope	–	–	Low	[73]
I239L	–	Yes	[90]	–	–
K78R	p10	Yes	[38]	No	[38]
KP177R	p22	Yes	[38]	Yes	[73]
B438L	p49	–	–	Yes	[73, 74]
E199L	j18L	–	–	Yes	[73]
O61R	p12	–	–	Yes	[73]
**Non-structural**					
A179L	–	–	–	Low	[73]
B119L	9GL, virus assembly	–	–	Yes	[74]
C475L	Poly(A) polymerase	Yes	[38]	–	–
C962R	DNA primase	Yes	[38]	–	–
DP71L	Protein phosphatase 1 regulator	–	–	Low	[73]
E165R	dUTPase	Yes	[38]	Yes	[38]
E296R	AP endonuclease	Yes	[38]	–	–
F1055L	Helicase	–	–	Yes	[73]
F334L	RNA reductase	Yes	[38]	–	–
G1211R	DNA polymerase	–	–	Yes	[73]
G1340L	VACV A7 early transcription factor large subunit-like	Yes	[86]	Low	[73]
H339R	Alpha-NAC binding protein	Yes	[38]	–	–
H359L	RNA polymerase subunit 3–11	Yes	[38]	–	–
I215L	Ubiquitin conjugating enzyme	Yes	[38]	Yes	[38]
I329L	TLR inhibitor	–	–	Low	[73]
NP1450L	RNA polymerase subunit 1	–	–	Yes	[73]
NP419L	DNA ligase	Yes	[38]	Yes	[73]
NP868R	mRNA-capping enzyme	Yes	[38]	–	–
O174L	DNA polymerase X	Yes	[38]	–	–
**Multigene families**					
MGF110-1 L	–	Yes	[38]	Yes	[38]
MGF110-2 L	–	–	–	Low	[73]
MGF110-4 L	XP124L	Yes	[38]	Yes	[38]
MGF110-5 L	V82L	Yes	[38]	Yes	[38]
MGF300-1 L	J268L	Yes	[38]	Low	[73]
MGF300-2R	–	–	–	Low	[73]
MGF360-11 L	KP362L	–	–	Yes	[73]
MGF360-15R	–	–	–	Low	[73]
MGF360-16R	–	Yes	[38]	–	–
MGF360-18R	–	–	–	Low	[73]
MGF360-1 L	KP360L	–	–	Yes	[73]
MGF505-11 L	–	Yes	[38]	–	–
MGF505-4R	–	–	–	Yes	[73]
MGF505-5R	A498R			Yes	[73]
**Uncharacterized**					
285 L	–	Yes	[38]	–	–
A151R	–	Yes	[38]	Yes	[38]
B407L	–	–	–	Low	[73]
C129R	–	Yes	[38]	Yes	[38]
C257L	–	Yes	[38]		
CP312R	–	Yes	[38]	Yes	[38]
CP312R	–	–	–	Yes	[31, 91]
D339L	–	–	–	Low	[73]
DP238L	–	Yes	[38]	–	–
E120R	–	–	.	Low	[73]
E146L	–	Yes	[38]	Yes	[38]
E184L	–	Yes	[38]	Yes	[38]
EP364R	–	–	–	Yes	[73]
F317L	–	–	–	Yes	[73]
I243L	–	Yes	[38]	–	–
I73R	–	Yes	[38]	No	[38]
K205R	–	Yes	[38]	Yes	[73, 74]
L8L	–	Yes	[38]	–	–
L10L	–	Yes	[38]	Low	[38, 73]
M1249L	–	Yes	[38]	Low	[73]
M448R	–	Yes	[38]	Yes	[38]
Complement (183875–184,183)	–	–	–	Low	[73]

The first ASFV CTL target identified in vitro was protein p32 [87], followed by the major capsid protein p72 and G1340L [86, 92]. Aiming to maximize the screening coverage of ASFV antigens, a random plasmid library of ASFV genomic DNA was generated and expressed in pig-derived fibroblasts. Sequencing of the clones that stimulated CD8^+^ T-cell proliferation revealed that one of the clones encoded a segment of I329L, a putative ASFV membrane protein. Notably, this technique allowed the identification of sequences that were in different reading frames than any of the known ASFV ORFs [90], indicating that the proteome of ASFV could be more complex than previously believed. This hypothesis has been recently supported by the identification of novel ASFV ORFs by RNA sequencing methods [93].

Despite the unarguable reality of CD8^+^ T-cells playing a role in protection, few ASFV antigens have demonstrated their protective potential (Table 4), still far from that afforded by LAVs. Today we know that CD2v can also exhort its protective effects by stimulating the induction of specific CD8^+^ T-cells [88, 89]. Thus, DNA immunization with a plasmid encoding p32, p54 and the extracellular domain of CD2v, fused to ubiquitin aiming to enhance the CTL responses induced, conferred partial protection against lethal challenge with E75. The protection afforded correlated with the induction of specific CD8^+^ T-cells recognizing two 9-mer peptides from the CD2v and in the absence of antibodies [89]. Work performed in our laboratory using DNA immunization has allowed not only the identification of new protective antigens but has also contributed to dissect the mechanisms involved in protection. Thus, immunization with a random library containing more than 4000 clones encoding random ASFV genome fragments (excluding the sequences encoding p32, p54 and CD2v to avoid competition) fused to ubiquitin protected 60% of the animals against E75 lethal challenge. The protection was again afforded in the absence of detectable specific antibodies after immunization and correlating with the induction of ASFV-specific T-cell responses [91] against several antigens, including CP312R [94]. These results clearly demonstrated the presence of additional protective CD8^+^ T-cell determinants within the ASFV genome. These results highlighted the convenience of formulating vaccines *a la carte*, encoding not only the appropriate ASFV antigens but also targeting them to specific antigen presentation pathways aiming to enhance the induction of protective responses and avoiding undesired adverse immune responses after vaccination [36].

**Table 4:** ASFV subunit vaccines tested in vivo that induced some level of protection

Gene Name	Immunization	Challenge strain	Outcome	Haplotype	Reference
A179L, B407L, B438L, B602L, B646L, Complement (183875–184,183), CP204L, CP2475L, CP530R, D117L, D339L, DP71L, E120R, E183L, E199L, EP153R, EP364R, EP402R, F1055L, F317L, G1211R, G1340L, H108R, I329L, K205R, KP177R, L10L, M1249L, MGF110-2 L, MGF110-4 L, MGF300-2R, MGF360-11 L, MGF360-15R, MGF360-18R, MGF360-1 L, MGF505-4R, MGF505-5R, NP1450L, NP419L & O61R	DNA prime + rVACV boost	Georgia 2007/1	No surviving pigs Reduced viremia and viral loads in some tissues	Babraham	[73]
B646L, CP204L, CP2475L (p37, p150), CP530R & E183L	rAd	Georgia 2007/1	Partial protection: 5/9 surviving pigs versus 1/5 control pigs Reduced viremia depending on the adjuvant used Pigs recover from infection Authors claim that none of the immunogens conferred statistically significant protection	Outbred	[71, 81]
A151R, B646L, C129R, CP204L, CP530R, E146L, I215L, I73R, L8L, M448R, MGF110-4 L & MGF110-5 L	rAd prime + MVA boost	OUR T88/1	No surviving pigs 3/6 pigs with reduced and delayed clinical signs, 5/6 pigs with reduced viremia Reduced viral load in some tissues	Outbred	[38]
CP2475L & A137R	Synthetic peptides	E70	No surviving pigs Increased average survival Reduced mean viral titers	Outbred	[72]
CP204L & B646L	Synthetic peptides	E70	No surviving pigs Increased average survival Reduced mean viral titers	Outbred	[72]
CP204L, E183L & EP402	DNA (as a fusion with Ub)	E75	Partial protection: 2/6 surviving pis Delayed clinical signs and viremia Pigs recover from the infection	Outbred	[89]
CP312R	DNA expression library	E75	Partial protection: 6/10 surviving pigs Reduced virus titers in blood, shedding and lesions Pigs recover from the infection	Outbred	[91, 94]

More recently, an overlapping peptide library comprising almost 4000 20-mers peptides overlapping by 10 amino acids from 133 different ASFV ORFs, was used in in vitro stimulation protocols using PBMCs from ASFV-convalescent pigs to identify potential CD8^+^ T-cell targets [38]. The specific responses induced were measured in an IFNγ ELISpot assay, identifying peptides from 38 ASFV proteins in at least one of the tested pigs, including p72 and p32, but also many other uncharacterized proteins (Table 3).

Interestingly, immunization with adenoviruses encoding the structural proteins: p32, p54, pp62, p72 and pp220 in the presence of the ZTS-01 adjuvant induced lower antibody responses and slightly better protection (non-statistically significant), than with the BioMize 0226 adjuvant, reflecting most probably the differential innate immune responses induced by each adjuvant [81]. On the other hand, immunization with cocktails up to 47 antigens in a DNA priming and vaccinia virus-boosting regime, allowed the identification of novel T-cell antigens. Unfortunately, none of the pigs survived the lethal challenge (Table 4) albeit some pigs showed a significant reduction of virus titers in blood and lymph nodes [73].

Finally, priming pigs with a cocktail of replication deficient adenoviruses encoding 12 ASFV ORFs and boosting with same antigens encoded in MVA, allowed the confirmation of a collection of antigens as capable of inducing specific T-cell responses and to partially protect pigs from challenge with OURT88/1 (Table 4). Although all the animals had to be sacrificed before day 8 after challenge, three out of six pigs showed delayed onset of clinical signs as well as reduced viremia. Interestingly, immunization with a second collection of immunogens provoked the exacerbation of the disease, coinciding with the induction of weaker CD8^+^ T-cell responses [38].

As a practical observation, we confirm ASFV in vivo challenge as the only valid method for evaluating the protective potential of an experimental vaccine. Lack of correlates of ASFV protection and protective targets, precludes assuming the potency of a given vaccine formulation based on the immune response induced.

Although the use of subunit vaccines based in ASFV fragments overcome most of the risks inherent to LAVs, their use in the field is far from becoming a reality due to the low efficacy demonstrated so far. As mentioned above, lack of profound knowledge of the protection mechanisms together with the absence of known protective antigens have complicated the design of efficient subunit vaccines against ASFV. These are in our understanding the future directions in research and development:

1. **Correlates of protection.** Easy vaccines have already been designed. ASFV is a complex virus capable of inhibiting the pig immune system by interfering with multiple pathways. Understanding ASFV strengths will teach us about its weakness. Optimal vaccination should not only enhance protective responses but also avoid prejudicial immune responses. 
2. **Antigen discovery.** Both humoral and cellular responses can be protective. Identifying as many antigens and/or epitopes from the whole proteome is absolutely needed. This has to be performed in parallel with understanding the mechanisms involved in protection.
3. **Antigen delivery.** We need to know not only what to induce and with which antigen, but also how to deliver it in an efficient manner and at an affordable price. Vaccinology will dramatically change in the future with the invention of new expression vectors that should be approved by the safety agencies.
4. **Adjuvants.** Little attention is given to the need of novel and efficient adjuvants to formulate a la carte vaccines, capable of inducing exclusively what is desired. Adjuvants could be an essential tool to achieve this goal. 
5. **Others.** DIVA concept, bait-vaccines and cross-protection. Subunit vaccines against ASFV will be DIVA by themselves with many immunodominant antigens available for differential diagnostics. Cross-protection however is an issue difficult to tackle today since we need to identify first the mechanisms and the antigens involved. Similarly, bait-vaccines for oral immunization of wild pigs is an utopia today, at least for ASF-subunit vaccines, albeit it has been recently proposed to experimentally immunize wild boars with a naturally attenuated ASFV strain [41]. We should first identify the antigens to incorporate, and then know how to formulate them in order to afford significant protection against ASF. We are not close of having a subunit vaccine, but if we do not invest in research today, they will never become a reality.

## Conclusions

ASFV vaccines are closer than they appear. On one hand, several experimental prototypes based on LAVs are mature enough to be transferred to the private sector for registration and field trials. International agencies, governments, industry and academy should join together to discuss the opportunity of their implementation in very specific scenarios, taking into account the benefits versus the prejudices that the new LAVs can provide and the different sensitivity for the ASF problem around the globe.

Parallel efforts should be made in research and development of more efficacious and safer subunit vaccines. Today far from the reality, achieving protection levels similar to those afforded by LAVs will be challenging. Complex formulations using multiple ASFV antigens or antigen fragments, adjuvants and expression vectors can be envisioned as the future choice, based on knowledge and scientific evidence.

ASF is a global problem that we were incapable of stopping. We should learn from our mistakes and find global solutions.

## Data Availability

Not applicable.

## References

[1] Alejo A, Matamoros T, Guerra M, Andrés G (2018). A proteomic atlas of the African swine fever virus particle. J Virol.

[2] Liu S, Luo Y, Wang Y, Li S, Zhao Z, Bi Y (2019). Cryo-EM structure of the African swine fever virus. Cell Host Microbe.

[3] Andrés G, Charro D, Matamoros T, Dillard RS, Abrescia NGA (2020). The cryo-EM structure of African swine fever virus unravels a unique architecture comprising two icosahedral protein capsids and two lipoprotein membranes. J Biol Chem.

[4] Dixon LK, Islam M, Nash R, Reis AL (2019). African swine fever virus evasion of host defences. Virus Res.

[5] Plowright W, Parker J, Peirce MA (1969). African swine fever virus in ticks (Ornithodoros moubata, Murray) collected from animal burrows in Tanzania. Nature..

[6] Bastos ADS, Penrith M-L, Crucière C, Edrich JL, Hutchings G, Roger F (2003). Genotyping field strains of African swine fever virus by partial p72 gene characterisation. Arch Virol.

[7] Pikalo J, Zani L, Hühr J, Beer M, Blome S (2019). Pathogenesis of African swine fever in domestic pigs and European wild boar - lessons learned from recent animal trials. Virus Res.

[8] Arias M, Sánchez-Vizcaíno JM (2002). African swine fever eradication: the Spanish model. Trends in emerging viral infections of swine.

[9] Sánchez-Cordón PJ, Montoya M, Reis AL, Dixon LK (2018). African swine fever: a re-emerging viral disease threatening the global pig industry. Vet J.

[10] Rowlands RJ, Michaud V, Heath L, Hutchings G, Oura C, Vosloo W (2008). African swine fever virus isolate, Georgia, 2007. Emerg Infect Dis.

[11] Dixon LK, Sun H, Roberts H (2019). African swine fever. Antivir Res.

[12] Gavier-Widén D, Ståhl K, Dixon L (2020). No hasty solutions for African swine fever. Science..

[13] Woodland DL. Commentary: an urgent need for an African swine fever vaccine. Viral Immunol. 2020;33(2):71. 10.1089/vim.2019.29045.dlw 31808729

[14] Mallapaty S (2019). Spread﻿ of deadly pig virus in China hastens vaccine research. Nature..

[15] Arias M, de la Torre A, Dixon L, Gallardo C, Jori F, Laddomada A, et al. Approaches and perspectives for development of African swine fever virus vaccines. Vaccines. 2017;5(4):35. . 10.3390/vaccines5040035 PMC5748602 28991171

[16] Stone SS, Hess WR (1967). Antibody response to inactivated preparations of African swine fever virus in pigs. Am J Vet Res.

[17] Blome S, Gabriel C, Beer M (2014). Modern adjuvants do not enhance the efficacy of an inactivated African swine fever virus vaccine preparation. Vaccine..

[18] Korenkov D, Isakova-Sivak I, Rudenko L (2018). Basics of CD8 T-cell immune responses after influenza infection and vaccination with inactivated or live attenuated influenza vaccine. Expert Rev Vaccines.

[19] Martins C, Mebus C, Scholl T, Lawman M, Lunney J (1988). Virus-specific CTL in SLA-inbred swine recovered from experimental African swine fever virus (ASFV) infection. Ann N Y Acad Sci.

[20] Martins CL, Lawman MJ, Scholl T, Mebus CA, Lunney JK (1993). African swine fever virus specific porcine cytotoxic T cell activity. Arch Virol.

[21] Scholl T, Lunney JK, Mebus CA, Duffy E, Martins CL (1989). Virus-specific cellular blastogenesis and interleukin-2 production in swine after recovery from African swine fever. Am J Vet Res.

[22] Zuckermann FA, Husmann RJ (1996). Functional and phenotypic analysis of porcine peripheral blood CD4/CD8 double-positive T cells. Immunology..

[23] Oura CA, Denyer MS, Takamatsu H, Parkhouse RM (2005). In vivo depletion of CD8+ T lymphocytes abrogates protective immunity to African swine fever virus. J Gen Virol..

[24] Denyer MS, Wileman TE, Stirling CMA, Zuber B, Takamatsu H-H (2006). Perforin expression can define CD8 positive lymphocyte subsets in pigs allowing phenotypic and functional analysis of natural killer, cytotoxic T, natural killer T and MHC un-restricted cytotoxic T-cells. Vet Immunol Immunopathol.

[25] Onisk DV, Borca MV, Kutish G, Kramer E, Irusta P, Rock DL (1994). Passively transferred African swine fever virus antibodies protect swine against lethal infection. Virology..

[26] Wardley RC, Norley SG, Wilkinson PJ, Williams S (1985). The role of antibody in protection against African swine fever virus. Vet Immunol Immunopathol.

[27] Gómez-Puertas P, Rodríguez F, Oviedo JM, Ramiro-Ibáñez F, Ruiz-Gonzalvo F, Alonso C (1996). Neutralizing antibodies to different proteins of African swine fever virus inhibit both virus attachment and internalization. J Virol.

[28] Escribano JM, Galindo I, Alonso C (2013). Antibody-mediated neutralization of African swine fever virus: myths and facts. Virus Res.

[29] Ruiz-Gonzalvo F, Rodríguez F, Escribano JM (1996). Functional and immunological properties of the baculovirus-expressed hemagglutinin of African swine fever virus. Virology..

[30] Ruiz Gonzalvo F, Carnero ME, Caballero C, Martínez J (1986). Inhibition of African swine fever infection in the presence of immune sera in vivo and in vitro. Am J Vet Res.

[31] Takamatsu H-H, Denyer MS, Lacasta A, Stirling CMA, Argilaguet JM, Netherton CL (2013). Cellular immunity in ASFV responses. Virus Res.

[32] Moulton J, Coggins L (1968). Comparison of lesions in acute and chronic African swine fever. Cornell Vet.

[33] Slauson DO, Sanchez-Vizcaino JM (1981). Leukocyte-dependent platelet vasoactive amine release and immune complex deposition in African swine fever. Vet Pathol.

[34] Fernandez A, Perez J, de las Mulas Martin L, Carrasco L, Dominguez J, Sierra MA (1992). Localization of African swine fever viral antigen, swine IgM, IgG and C1q in lung and liver tissues of experimentally infected pigs. J Comp Pathol.

[35] Pan IC, Moulton JE, Hess WR (1975). Immunofluorescent studies on chronic pneumonia in swine with experimentally induced African swine fever. Am J Vet Res.

[36] Argilaguet JM, Pérez-Martín E, Gallardo C, Salguero FJ, Borrego B, Lacasta A (2011). Enhancing DNA immunization by targeting ASFV antigens to SLA-II bearing cells. Vaccine..

[37] Sunwoo S-Y, Pérez-Núñez D, Morozov I, Sánchez E, Gaudreault N, Trujillo J (2019). DNA-protein vaccination strategy does not protect from challenge with African swine fever virus Armenia 2007 strain. Vaccines..

[38] Netherton CL, Goatley LC, Reis AL, Portugal R, Nash RH, Morgan SB (2019). Identification and immunogenicity of African swine fever virus antigens. Front Immunol.

[39] Nurmoja I, Petrov A, Breidenstein C, Zani L, Forth JH, Beer M (2017). Biological characterization of African swine fever virus genotype II strains from North-Eastern Estonia in European wild boar. Transbound Emerg Dis.

[40] Gallardo C, Soler A, Rodze I, Nieto R, Cano-Gómez C, Fernandez-Pinero J (2019). Attenuated and non-haemadsorbing (non-HAD) genotype II African swine fever virus (ASFV) isolated in Europe, Latvia 2017. Transbound Emerg Dis.

[41] Barasona JA, Gallardo C, Cadenas-Fernández E, Jurado C, Rivera B, Rodríguez-Bertos A, et al. First oral vaccination of Eurasian wild boar against African swine fever virus genotype II. Front Vet Sci. 2019;6:137. 10.3389/fvets.2019.00137 PMC6498142 31106218

[42] Gallardo C, Nieto R, Soler A, Pelayo V, Fernández-Pinero J, Markowska-Daniel I (2015). Assessment of African swine fever diagnostic techniques as a response to the epidemic outbreaks in eastern european union countries: how to improve surveillance and control programs. J Clin Microbiol.

[43] Garcia R, Almazán F, Rodríguez JM, Alonso M, Viñuela E, Rodríguez JF (1995). Vectors for the genetic manipulation of African swine fever virus. J Biotechnol.

[44] Borca MV, Holinka LG, Berggren KA, Gladue DP (2018). CRISPR-Cas9, a tool to efficiently increase the development of recombinant African swine fever viruses. Sci Rep.

[45] Rodríguez JM, Yáñez RJ, Almazán F, Viñuela E, Rodriguez JF (1993). African swine fever virus encodes a CD2 homolog responsible for the adhesion of erythrocytes to infected cells. J Virol.

[46] Borca MV, Carrillo C, Zsak L, Laegreid WW, Kutish GF, Neilan JG (1998). Deletion of a CD2-like gene, 8-DR, from African swine fever virus affects viral infection in domestic swine. J Virol.

[47] Borca MV, O’Donnell V, Holinka LG, Risatti GR, Ramirez-Medina E, Vuono EA (2020). Deletion of CD2-like gene from the genome of African swine fever virus strain Georgia does not attenuate virulence in swine. Sci Rep.

[48] Monteagudo PL, Lacasta A, López E, Bosch L, Collado J, Pina-Pedrero S, et al. BA71ΔCD2: a new recombinant live attenuated African swine fever virus with cross-protective capabilities. J Virol. 2017;91(21):e01058-17. 10.1128/JVI.01058-17 PMC5640839 28814514

[49] Zsak L, Lu Z, Kutish GF, Neilan JG, Rock DL (1996). An African swine fever virus virulence-associated gene NL-S with similarity to the herpes simplex virus ICP34.5 gene. J Virol.

[50] Afonso CL, Zsak L, Carrillo C, Borca MV, Rock DL (1998). African swine fever virus NL gene is not required for virus virulence. J Gen Virol..

[51] Neilan JG, Zsak L, Lu Z, Kutish GF, Afonso CL, Rock DL (2002). Novel swine virulence determinant in the left variable region of the African swine fever virus genome. J Virol.

[52] Carlson J, O’Donnell V, Alfano M, Salinas LV, Holinka LG, Krug PW, et al. Association of the host immune response with protection using a live attenuated African swine fever virus model. Viruses. 2016;8(10):291. 10.3390/v8100291 PMC5086623 27782090

[53] Lewis T, Zsak L, Burrage TG, Lu Z, Kutish GF, Neilan JG (2000). An African swine fever virus ERV1-ALR homologue, 9GL, affects virion maturation and viral growth in macrophages and viral virulence in swine. J Virol.

[54] O’Donnell V, Holinka LG, Krug PW, Gladue DP, Carlson J, Sanford B (2015). African swine fever virus Georgia 2007 with a deletion of virulence-associated gene 9GL (B119L), when administered at low doses, leads to virus attenuation in swine and induces an effective protection against homologous challenge. J Virol.

[55] Zsak L, Caler E, Lu Z, Kutish GF, Neilan JG, Rock DL (1998). A nonessential African swine fever virus gene UK is a significant virulence determinant in domestic swine. J Virol.

[56] Reis AL, Goatley LC, Jabbar T, Sanchez-Cordon PJ, Netherton CL, Chapman DAG, et al. Deletion of the African swine fever virus gene DP148R does not reduce virus replication in culture but reduces virus virulence in pigs and induces high levels of protection against challenge. J Virol. 2017;15, 91(24):e01428-17. 10.1128/JVI.01428-17 PMC5709585 28978700

[57] Reis AL, Abrams CC, Goatley LC, Netherton C, Chapman DG, Sanchez-Cordon P (2016). Deletion of African swine fever virus interferon inhibitors from the genome of a virulent isolate reduces virulence in domestic pigs and induces a protective response. Vaccine..

[58] O’Donnell V, Holinka LG, Gladue DP, Sanford B, Krug PW, Lu X (2015). African swine fever virus Georgia isolate harboring deletions of MGF360 and MGF505 genes is attenuated in swine and confers protection against challenge with virulent parental virus. J Virol.

[59] O’Donnell V, Risatti GR, Holinka LG, Krug PW, Carlson J, Velazquez-Salinas L, et al. Simultaneous deletion of the 9GL and UK genes from the African swine fever virus Georgia 2007 isolate offers increased safety and protection against homologous challenge. J Virol. 2017;91(1):e01760-16. 10.1128/JVI.01760-16 PMC5165186 27795430

[60] O’Donnell V, Holinka LG, Sanford B, Krug PW, Carlson J, Pacheco JM (2016). African swine fever virus Georgia isolate harboring deletions of 9GL and MGF360/505 genes is highly attenuated in swine but does not confer protection against parental virus challenge. Virus Res.

[61] Ramirez-Medina E, Vuono E, O’Donnell V, Holinka LG, Silva E, Rai A, et al. Differential effect of the deletion of African swine fever virus virulence-associated genes in the induction of attenuation of the highly virulent Georgia strain. Viruses. 2019;11(7):599. 10.3390/v11070599 PMC6669436 31269702

[62] Abrams CC, Goatley L, Fishbourne E, Chapman D, Cooke L, Oura CA (2013). Deletion of virulence associated genes from attenuated African swine fever virus isolate OUR T88/3 decreases its ability to protect against challenge with virulent virus. Virology..

[63] Gallardo C, Sánchez EG, Pérez-Núñez D, Nogal M, de León P, Carrascosa ÁL (2018). African swine fever virus (ASFV) protection mediated by NH/P68 and NH/P68 recombinant live-attenuated viruses. Vaccine..

[64] Chen W, Zhao D, He X, Liu R, Wang Z, Zhang X, et al. A seven-gene-deleted African swine fever virus is safe and effective as a live attenuated vaccine in pigs. Sci China Life Sci. 2020;63(5):623-34. 10.1007/s11427-020-1657-9 PMC7223596 32124180

[65] Salas ML, Andrés G (2013). African swine fever virus morphogenesis. Virus Res.

[66] Rodríguez JM, Moreno LT, Alejo A, Lacasta A, Rodríguez F, Salas ML. Genome sequence of african swine fever virus BA71, the virulent parental strain of the nonpathogenic and tissue-culture adapted BA71V. PLoS One. 2015;10(11):e0142889. 10.1371/journal.pone.0142889 PMC4664411 26618713

[67] Malogolovkin A, Burmakina G, Titov I, Sereda A, Gogin A, Baryshnikova E (2015). Comparative analysis of African swine fever virus genotypes and serogroups. Emerg Infect Dis.

[68] Borca MV, Ramirez-Medina E, Silva E, Vuono E, Rai A, Pruitt S, et al. Development of a highly effective African swine fever virus vaccine by deletion of the I177L gene results in sterile immunity against the current epidemic Eurasia strain. J Virol. 2020;94(7):e02017-19. 10.1128/JVI.02017-19 PMC7081903 31969432

[69] Kollnberger SD, Gutierrez-Castañeda B, Foster-Cuevas M, Corteyn A, Parkhouse RM (2002). Identification of the principal serological immunodeterminants of African swine fever virus by screening a virus cDNA library with antibody. J Gen Virol..

[70] Gallardo C, Reis AL, Kalema-Zikusoka G, Malta J, Soler A, Blanco E (2009). Recombinant antigen targets for serodiagnosis of African swine fever. Clin Vaccine Immunol.

[71] Lokhandwala S, Waghela SD, Bray J, Martin CL, Sangewar N, Charendoff C (2016). Induction of robust immune responses in swine by using a cocktail of adenovirus-vectored African swine fever virus antigens. Clin Vaccine Immunol.

[72] Ivanov V, Efremov EE, Novikov BV, Balyshev VM, Tsibanov SZ, Kalinovsky T (2011). Vaccination with viral protein-mimicking peptides postpones mortality in domestic pigs infected by African swine fever virus. Mol Med Rep.

[73] Jancovich JK, Chapman D, Hansen DT, Robida MD, Loskutov A, Craciunescu F (2018). Immunization of pigs by DNA prime and recombinant Vaccinia virus boost to identify and rank African swine fever virus immunogenic and protective proteins. J Virol.

[74] Lokhandwala S, Waghela SD, Bray J, Sangewar N, Charendoff C, Martin CL (2017). Adenovirus-vectored novel African swine fever virus antigens elicit robust immune responses in swine. PLoS One.

[75] Gutiérrez-Castañeda B, Reis AL, Corteyn A, Parkhouse RME, Kollnberger S (2008). Expression, cellular localization and antibody responses of the African swine fever virus genes B602L and K205R. Arch Virol.

[76] Zsak L, Onisk DV, Afonso CL, Rock DL (1993). Virulent African swine fever virus isolates are neutralized by swine immune serum and by monoclonal antibodies recognizing a 72-kDa viral protein. Virology..

[77] Neilan JG, Zsak L, Lu Z, Burrage TG, Kutish GF, Rock DL (2004). Neutralizing antibodies to African swine fever virus proteins p30, p54, and p72 are not sufficient for antibody-mediated protection. Virology..

[78] Pérez-Núñez D, Sunwoo SY, Sánchez EG, Haley N, García-Belmonte R, Nogal M (2019). Evaluation of a viral DNA-protein immunization strategy against African swine fever in domestic pigs. Vet Immunol Immunopathol.

[79] Barderas MG, Rodríguez F, Gómez-Puertas P, Avilés M, Beitia F, Alonso C (2001). Antigenic and immunogenic properties of a chimera of two immunodominant African swine fever virus proteins. Arch Virol.

[80] Gallardo C, Blanco E, Rodríguez JM, Carrascosa AL, Sanchez-Vizcaino JM (2006). Antigenic properties and diagnostic potential of African swine fever virus protein pp62 expressed in insect cells. J Clin Microbiol.

[81] Lokhandwala S, Petrovan V, Popescu L, Sangewar N, Elijah C, Stoian A (2019). Adenovirus-vectored African swine fever virus antigen cocktails are immunogenic but not protective against intranasal challenge with Georgia 2007/1 isolate. Vet Microbiol.

[82] Ruíz-Gonzalvo F, Coll JM (1993). Characterization of a soluble hemagglutinin induced in African swine fever virus-infected cells. Virology..

[83] Burmakina G, Malogolovkin A, Tulman ER, Zsak L, Delhon G, Diel DG (2016). African swine fever virus serotype-specific proteins are significant protective antigens for African swine fever. J Gen Virol..

[84] Argilaguet JM, Pérez-Martín E, López S, Goethe M, Escribano JM, Giesow K (2013). BacMam immunization partially protects pigs against sublethal challenge with African swine fever virus. Antivir Res.

[85] Reis AL, Parkhouse RME, Penedos AR, Martins C, Leitao A (2007). Systematic analysis of longitudinal serological responses of pigs infected experimentally with African swine fever virus. J Gen Virol..

[86] Leitão A, Malur A, Cartaxeiro C, Vasco G, Cruz B, Cornelis P (2000). Bacterial lipoprotein based expression vectors as tools for the characterisation of African swine fever virus (ASFV) antigens. Arch Virol.

[87] Alonso F, Domínguez J, Viñuela E, Revilla Y (1997). African swine fever virus-specific cytotoxic T lymphocytes recognize the 32 kDa immediate early protein (vp32). Virus Res.

[88] Burmakina G, Malogolovkin A, Tulman ER, Xu W, Delhon G, Kolbasov D (2019). Identification of T-cell epitopes in African swine fever virus CD2v and C-type lectin proteins. J Gen Virol.

[89] Argilaguet JM, Pérez-Martín E, Nofrarías M, Gallardo C, Accensi F, Lacasta A, et al. DNA vaccination partially protects against African swine fever virus lethal challenge in the absence of antibodies. PLoS One. 2012;7(9):e40942. 10.1371/journal.pone.0040942 PMC3458849 23049728

[90] Jenson JS, Childerstone A, Takamatsu H, Dixon LK, Parkhouse RM (2000). The cellular immune recognition of proteins expressed by an African swine fever virus random genomic library. J Immunol Methods.

[91] Lacasta A, Ballester M, Monteagudo PL, Rodríguez JM, Salas ML, Accensi F (2014). Expression library immunization can confer protection against lethal challenge with African swine fever virus. J Virol.

[92] Leitão A, Malur A, Cornelis P, Martins CL (1998). Identification of a 25-aminoacid sequence from the major African swine fever virus structural protein VP72 recognised by porcine cytotoxic T lymphocytes using a lipoprotein based expression system. J Virol Methods.

[93] Cackett G, Matelska D, Sýkora M, Portugal R, Malecki M, Bähler J, et al. The African swine fever virus transcriptome. J Virol. 2020;94(9):e00119-20. 10.1128/JVI.00119-20 PMC7163114 32075923

[94] Lacasta A. Nuevos avances en el desarrollo de vacunas frente a la peste porcina africana [PhD dissertation]. Barcelona: Universitat de Barcelona; 2012.

